# Disrupting the Electrical Circuit: New Onset Atrial Fibrillation in a Patient With Severe Acute Respiratory Syndrome Coronavirus 2 (SARS-CoV-2)

**DOI:** 10.7759/cureus.9082

**Published:** 2020-07-09

**Authors:** Sohab Radwan, Owen Schwartz

**Affiliations:** 1 Internal Medicine, MedStar Washington Hospital Center, Washington, DC, USA; 2 Internal Medicine/Hospital Medicine, MedStar Washington Hospital Center, Washington, DC, USA

**Keywords:** sars-cov-2, covid-19, arrhythmias, atrial fibrillation

## Abstract

In December 2019, an outbreak of pneumonia cases in Wuhan, China was attributed to a novel coronavirus that was eventually recognized as severe acute respiratory syndrome coronavirus 2 (SARS-CoV-2). Currently identified as coronavirus disease 2019 (COVID-19), it has been declared a pandemic by the World Health Organization given its rapid global transmission. Various cardiovascular complications have been reported, including heart failure, myocarditis, acute coronary syndrome and arrhythmias, both atrial and ventricular. Regarding arrhythmias, onset from time of infection is variable but usually ranges from several days to a week. We hereby present a case of a COVID-19 positive patient presenting with new onset atrial fibrillation.

## Introduction

In December 2019, a cluster of cases of pneumonia of unknown cause were identified in Wuhan, Hubei, China that was eventually attributed to a novel enveloped coronavirus, currently named as severe acute respiratory syndrome coronavirus 2 (SARS-CoV-2) [1]. Now identified as coronavirus disease 2019 (COVID-19), the World Health Organization has declared SARS-CoV-2 a public health emergency and pandemic given its rapid spread across several countries worldwide [2]. Several cardiovascular manifestations have been reported so far, including arrhythmias, heart failure, cardiogenic shock, fulminant myocarditis and acute coronary syndrome [3]. Several reports worldwide have demonstrated a high incidence of arrhythmias, specifically supraventricular, in the setting of widespread and systemic inflammation associated with COVID-19. The prevalence of arrhythmias and conduction system disease in patients with COVID-19 varies from population to population [4]. Several case reports have shown that onset of atrial fibrillation may vary from a few days up to a week from acquiring the infection; however, sometimes may be longer [5,6]. Regarding the mechanism, hypoxia and electrolyte abnormalities, both known to contribute to the development of acute arrhythmias, have been frequently reported in the acute phase of COVID-19 illness [4]. We hereby present a case of a COVID-19 positive patient presenting with new onset atrial fibrillation and shed light on the current literature and the proposed mechanisms and etiology.

## Case presentation

**Figure 1:**
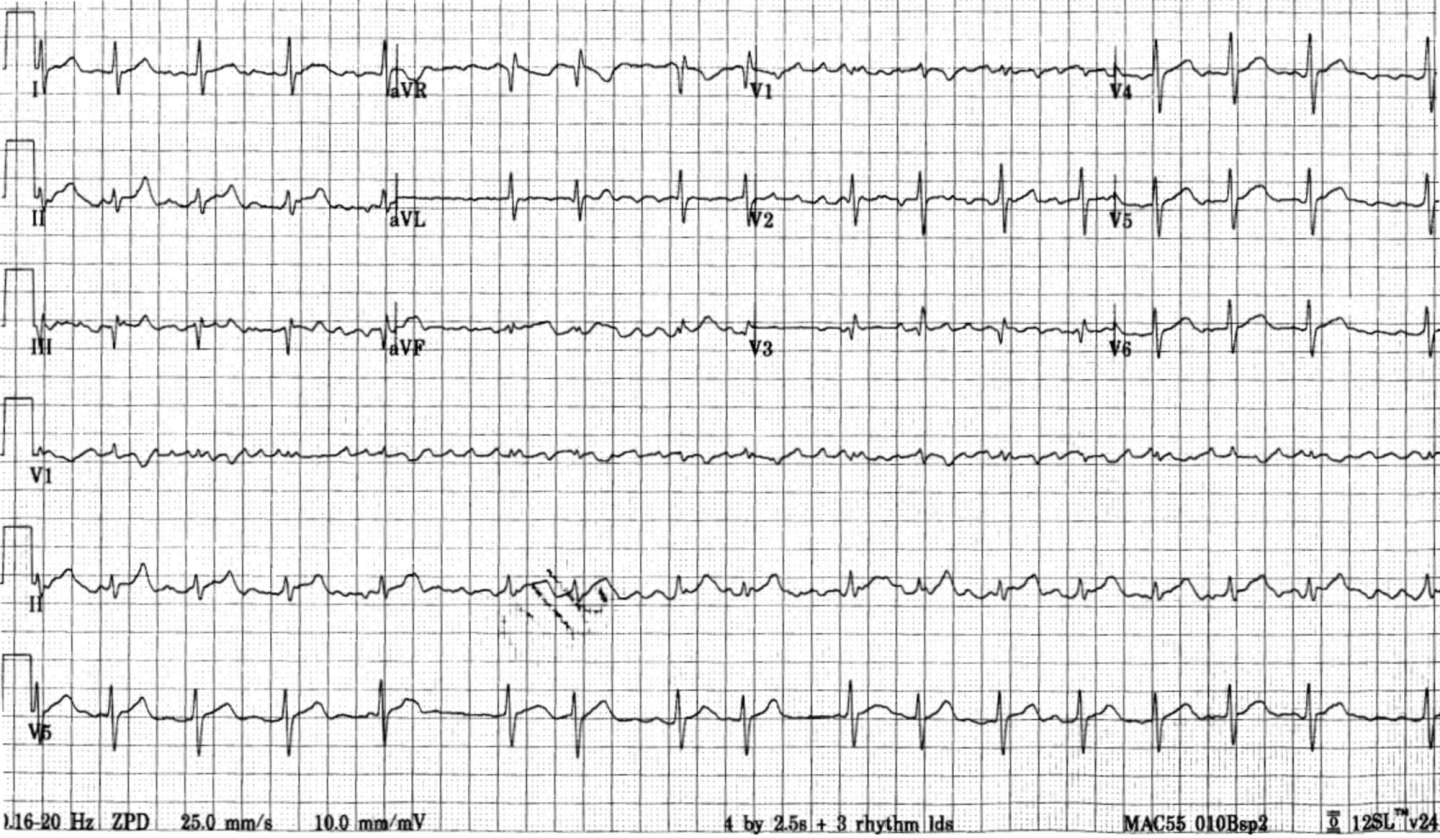
A 12-lead electrocardiogram demonstrating atrial fibrillation, calculated heart rate of 102 beats per minute, normal axis, no significant ST-segment deviation and non-specific T-wave abnormalities.

A 37-year-old male patient with no significant past medical history presented to the emergency department with left foot pain of one-day duration following direct trauma. No other symptoms were reported on presentation, including palpitations, chest pain, shortness of breath or dizziness. Of importance, the patient reports multiple family members testing positive for SARS-CoV-2 two weeks ago as well as himself having experienced mild flu-like symptoms several days ago. Regarding social history, the patient denied any current or prior tobacco smoking, alcohol consumption or other drug abuse. Initial vital signs were notable for heart rate of 90 beats per minute, blood pressure of 152/89 mmHg, respiratory rate of 20 breaths per minute and an oxygen saturation of 100% on room air. The patient's weight is 86 kilograms with a calculated body mass index of 26.8. A 12-lead electrocardiogram demonstrated an irregularly irregular rhythm consistent with atrial fibrillation (Figure 1).

No baseline electrocardiogram was present in the medical chart. Pertinent diagnostic laboratory investigations demonstrated a normal level of troponin I, thyroid-stimulating hormone, hemoglobin and electrolytes, including potassium, magnesium and calcium (Table 1).

**Table 1:** Initial laboratory values SARS-CoV-2: severe acute respiratory syndrome coronavirus 2; RNA: ribonucleic acid; PCR: polymerase chain reaction

Test	Value	Reference Range
Cardiac testing		
Troponin I (ng/mL)	<0.017	0.000–0.045
General chemistry		
Sodium (mmol/L)	139	137–145
Potassium (mmol/L)	4.1	3.5–5.1
Chloride (mmol/L)	105	98–107
Sodium bicarbonate (mmol/L)	21	21–32
Blood urea nitrogen (mg/dL)	11	9–20
Creatinine (mg/dL)	0.77	0.66–1.50
Glucose (mg/dL)	131	65–140
Calcium (mg/dL)	9.0	8.5–10.1
Magnesium (mg/dL)	2.1	1.6–2.3
Hematology		
White blood cell count (k/µL)	8.4	4.0–10.8
Hemoglobin (g/dL)	14.7	12.5–16.5
Hematocrit (%)	43.5	37.5–49.5
Platelets (k/µL)	303	145–400
Endocrinology		
Thyroid-stimulating hormone (uIU/mL)	1.150	0.400–4.000
Infectious disease		
SARS-CoV-2 RNA PCR	Positive	Negative

Initial evaluation by the podiatric surgery team revealed a left fifth proximal phalanx displaced fracture. Decision was to take the patient to the operating theater for open reduction internal fixation of this fracture. Per our hospital's policy for surgical procedures, a SARS-CoV-2 polymerase chain reaction test was done and resulted as positive. He eventually underwent the procedure without any complications. Unfortunately, no echocardiogram was obtained to investigate the presence of structural heart disease. No rate control agents were required throughout his hospitalization. CHA2DS2-VASc (congestive heart failure, hypertension, age ≥75 years [double score], diabetes, prior stroke or transient ischemic attack [TIA; double score], vascular disease, age 65-74 years, sex class [female]) score for atrial fibrillation was zero, and therefore anticoagulation was not indicated. The patient was eventually discharged home with a new diagnosis of atrial fibrillation. Despite it being difficult to establish a causation between COVID-19 and atrial fibrillation in our patient, especially in the absence of an echocardiogram, this report aims to highlight the possible association between those two conditions.

## Discussion

Cardiac arrhythmias have been reported in several recent reports in the literature in relation to hospitalized patients with COVID-19. Additionally, it was demonstrated to be associated with a higher risk of mortality [7]. However, the exact arrhythmic risk related to COVID-19 in patients with less severe illness or those who recover from the acute phase of the severe illness is currently unknown [4]. In regards to incidence, a report on 138 hospitalized COVID-19 patients in China has shown that 16.7% of patients developed cardiac arrhythmias [8]. Unfortunately, the exact nature of those cardiac arrhythmias was not reported in this study. However, given that sepsis in general has been associated with an increased rate of atrial arrhythmias, one would expect that those associated with COVID-19 to be mostly atrial as well [9]. Different types of arrhythmias have been reported so far including atrial fibrillation, atrial flutter, ventricular tachycardia as well as ventricular fibrillation. Interestingly, one report showed that arrhythmias were observed in 7% of patients who did not require intensive care unit treatment and in 44% of those who did [10].

Regarding pathophysiology, multiple mechanisms exist in which COVID-19 can trigger cardiac arrhythmias. Potential contributing factors include metabolic derangements, acidosis and hypoxia [11]. Moreover, the associated neurohormonal and catecholaminergic stress is thought to play a major role [11]. Similar to sepsis, the inflammatory cytokines and autonomic dysfunction associated with COVID-19 is thought to be a significant trigger for arrhythmias, especially atrial ones. Specifically, atrial fibrillation as a sequela of critical illness has been reported to occur in almost 10% of intensive care unit patients [12]. In addition, supraventricular tachyarrhythmias such as atrial fibrillation are associated more frequently with sympathetic nervous system activity in comparison to ventricular ones. Consequently, this explains the high prevalence of supraventricular tachyarrhythmias in patients with COVID-19 given the associated hyperbolic sympathetic activity [13].

It is noteworthy to mention that malignant cardiac arrhythmias, such as sustained ventricular tachycardia and ventricular fibrillation, have been observed in hospitalized patients with COVID-19. Despite the limited clinical and published data, this most likely occurs in the setting of COVID-19-associated acute cardiac injury and myocarditis [14]. Moreover, patients with inherited arrhythmia syndromes, such as long QT syndrome, may be at an increased proarrhythmic risk in the setting of COVID-19 infection, necessitating specialized care [15]. Improved understanding of this issue is critical, and further research is needed in guiding the need for additional arrhythmia monitoring post discharge and whether cardioverter defibrillators will be required in those with impaired left ventricular function attributed to COVID-19 [4].

## Conclusions

In light of the emerging pandemic of COVID-19, several cardiovascular complications including arrhythmias are gaining worldwide recognition. Awareness of such entities and their theoretical pathophysiology is required for early recognition. Future research is needed to further investigate those observations and the associated clinical outcomes.
